# Supplementary material for: Can Baseline IL-6 Levels Predict Long COVID in Subjects Hospitalized for SARS-CoV-2 Disease?
Source: Int J Mol Sci. 2023 Jan 15;24(2):1731. doi: 10.3390/ijms24021731 (PMC9866804; doi:10.3390/ijms24021731)
Supplement: Supplementary file 1 [file ijms-24-01731-s001.zip › ijms-2101583-supplementary.pdf]

Table S1. Association between baseline serum IL-6 levels and long COVID symptomatology

Sign or symptom	% of cases in normal serum IL-6 levels	% of cases in high serum IL-6 levels	Model 1 (OR, 95% CI; p-value)	Model 2 (OR, 95% CI; p-value)	Model 3 (OR, 95% CI; p-value)
Any	35.4	64.2	1.54 (1.08-3.03) P=0.02	1.56 (1.07-3.19) P=0.02	2.05 (1.04-4.50) p=0.03
Headache	5.2	4.9	0.98 (0.21-4.69) P=0.98	0.88 (0.15-5.10) P=0.88	0.74 (0.10-5.20) P=0.76
Taste disorder (ageusia or dysgeusia)	5.2	4.9	0.66 (0.14-3.12) P=0.60	0.71 (0.13-3.93) P=0.69	1.10 (0.14-8.59) P=0.93
Smell disorder (anosmia)	3.4	3.3	1.09 (0.19-6.35) P=0.93	0.69 (0.09-5.32) P=0.72	0.50 (0.05-5.31) P=0.57
Memory deficits	22.4	26.2	1.40 (0.62-3.18) P=0.38	1.52 (0.65-3.57) P=0.34	1.67 (0.64-4.36) P=0.29
Difficulty concentrating	17.2	24.6	1.61 (0.68-3.82) P=0.28	1.60 (0.65-3.91) P=0.31	1.75 (0.66-4.66) P=0.26
Dizziness	6.9	4.9	0.76 (0.19-2.94) P=0.69	0.68 (0.16-2.98) P=0.61	2.29 (0.33-16.02) P=0.41
Tremors	5.2	3.3	0.49 (0.10-2.57) P=0.40	0.48 (0.09-2.58) P=0.39	0.27 (0.04-2.02) P=0.20
Cramps	6.9	3.3	0.36 (0.08-1.72) P=0.20	0.25 (0.04-1.39) P=0.11	0.17 (0.02-1.31) P=0.09
Visual impairment	6.9	4.1	1.31 (0.24-7.14) P=0.76	1.32 (0.23-7.58) P=0.76	3.66 (0.41-32.57) P=0.25
Cough	6.9	7.4	1.25 (0.30-2.26) P=0.76	1.67 (0.37-7.47) P=0.50	2.25 (0.38-13.31) P=0.37
Dyspnea	24.1	20.5	0.77 (0.34-1.74) P=0.53	0.86 (0.36-2.07) P=0.74	1.16 (0.44-3.04) P=0.76
Oxygen use	1.7	4.1	2.04 (0.22-18.9) P=0.53	1.74 (0.13-21.49) P=0.68	1.38 (0.09-21.9) P=0.82
Nasal congestion	3.4	1.6	0.49 (0.07-3.63) P=0.49	0.35 (0.04-3.07) P=0.34	0.46 (0.04-5.89) P=0.55
Voice change	3.4	2.5	0.81 (0.12-5.21) P=0.82	1.74 (0.15-20.44) P=0.66	2.65 (0.12-50.91) P=0.56
Mobility decline	6.9	13.1	2.02	2.40	2.55

Sign or symptom	% of cases in normal serum IL-6 levels	% of cases in high serum IL-6 levels	Model 1 (OR, 95% CI; p-value)	Model 2 (OR, 95% CI; p-value)	Model 3 (OR, 95% CI; p-value)
			(1.63-651) P=0.02	(1.71-8.11) P=0.02	(1.08-9.40) p=0.02
Palpitations	10.3	5.7	0.62 (0.16-2.46) P=0.49	0.62 (0.15-2.62) P=0.52	0.79 (0.14-4.54) P=0.80
Chest pain	8.6	4.1	0.39 (0.10-1.56) P=0.18	0.31 (0.06-1.53) P=0.15	0.88 (0.10-8.16) P=0.91
Flushing	0.0	2.5	Not possible	Not possible	Not possible
Abdominal pain	5.2	0.8	0.23 (0.02-2.68) P=0.24	0.25 (0.02-2.97) P=0.27	0.23 (0.01-3.77) P=0.30
Diarrhea	1.7	2.5	1.68 (0.16-17.23) P=0.66	2.36 (0.20-29.98) P=0.50	2.39 (0.16-35.87) P=0.53
Vomit	1.7	0.0	Not possible	Not possible	Not possible
Loss of appetite	6.9	1.6	0.48 (0.07-3.53) P=0.47	0.05 (0.001-2.63) P=0.14	0.23 (0.001-107.4) P=0.64
Rash	3.4	2.5	1.38 (0.14-13.68) P=0.79	1.53 (0.14-16.54) P=0.73	1.09 (0.08-15.80) P=0.95
Hair loss	6.9	4.9	1.03 (0.21-5.17) P=0.97	1.00 (0.19-5.39) P=0.99	0.30 (0.03-2.74) P=0.29
Involuntary weight loss	3.4	1.6	0.48 (0.07-3.53) P=0.47	0.40 (0.05-3.29) P=0.40	0.24 (0.02-3.03) P=0.27
Myalgia	20.7	17.2	1.05 (0.43-2.56) P=0.91	1.03 (0.41-2.59) P=0.95	1.30 (0.46-3.68) P=0.62
Pain	22.8	19.7	0.92 (0.41-2.09) P=0.84	0.96 (0.41-2.24) P=0.93	1.20 (0.47-3.08) P=0.70
Flulike symptoms	3.4	3.3	1.10 (0.18-6.56) P=0.92	1.12 (0.17-7.19) P=0.91	1.47 (0.18-11.91) P=0.72
Fever	0.0	1.7	Not possible	Not possible	Not possible
Fatigue	43.1	40.2	1.08 (0.54-2.14) P=0.83	1.12 (0.54-2.31) P=0.76	1.55 (0.70-3.46) P=0.28
Arthralgia	20.7	17.2	1.12 (0.46-2.69) P=0.80	1.32 (0.53-3.27) P=0.56	1.83 (0.66-5.07) P=0.25
Sore throat	5.2	2.5	0.49 (0.09-2.60) P=0.40	0.40 (0.07-2.39) P=0.31	0.22 (0.02-2.14) P=0.19
Sweats	6.9	1.6	0.34	0.30	0.24

Sign or symptom	% of cases in normal serum IL-6 levels	% of cases in high serum IL-6 levels	Model 1 (OR, 95% CI; p-value)	Model 2 (OR, 95% CI; p-value)	Model 3 (OR, 95% CI; p-value)
			(0.05-2.23) P=0.26	(0.04-2.40) P=0.25	(0.02-3.14) P=0.27
Conjunctivitis	5.2	0.8	0.16 (0.02-1.55) P=0.11	0.13 (0.01-1.49) P=0.10	0.07 (0.002-2.84) P=0.16
PTSD	5.5	12.3	1.86 (0.57-6.11) P=0.31	2.00 (1.05-6.69) P=0.03	2.38 (1.06-8.61) p=0.02
Depression	11.3	10.8	0.76 (0.26-2.22) P=0.62	0.78 (0.26-2.33) P=0.65	1.52 (0.40-5.72) P=0.54
Sleep disorders	17.2	11.5	0.93 (0.34-2.55) P=0.88	0.81 (0.28-2.39) P=0.71	1.25 (0.35-4.42) P=0.73
Anxiety	10.7	15.4	2.00 (0.62-6.38) P=0.24	2.05 (0.63-6.66) P=0.24	2.66 (0.77-9.20) P=0.12

All the data are reported as odds ratio with 95% confidence interval and correspondent p-values, calculated for elevated serum IL-6 vs. normal values. Model 1 was adjusted for age and sex; model 2 was adjusted for covariates in model 1 and comorbidities (yes vs. no), smoking status (actual, previous, never), PaO2/FiO2 ratio, haemoglobin levels, renal function (all measured within the first four days of hospitalization), presence of pneumonia during hospitalization; model 3 was adjusted for the covariates included in model 1 and model 2 and other serum parameters of inflammation (white cells, C reactive protein, changes during hospitalization of IL-6).

Figure S1. Distribution of serum IL-6 levels in the patients included

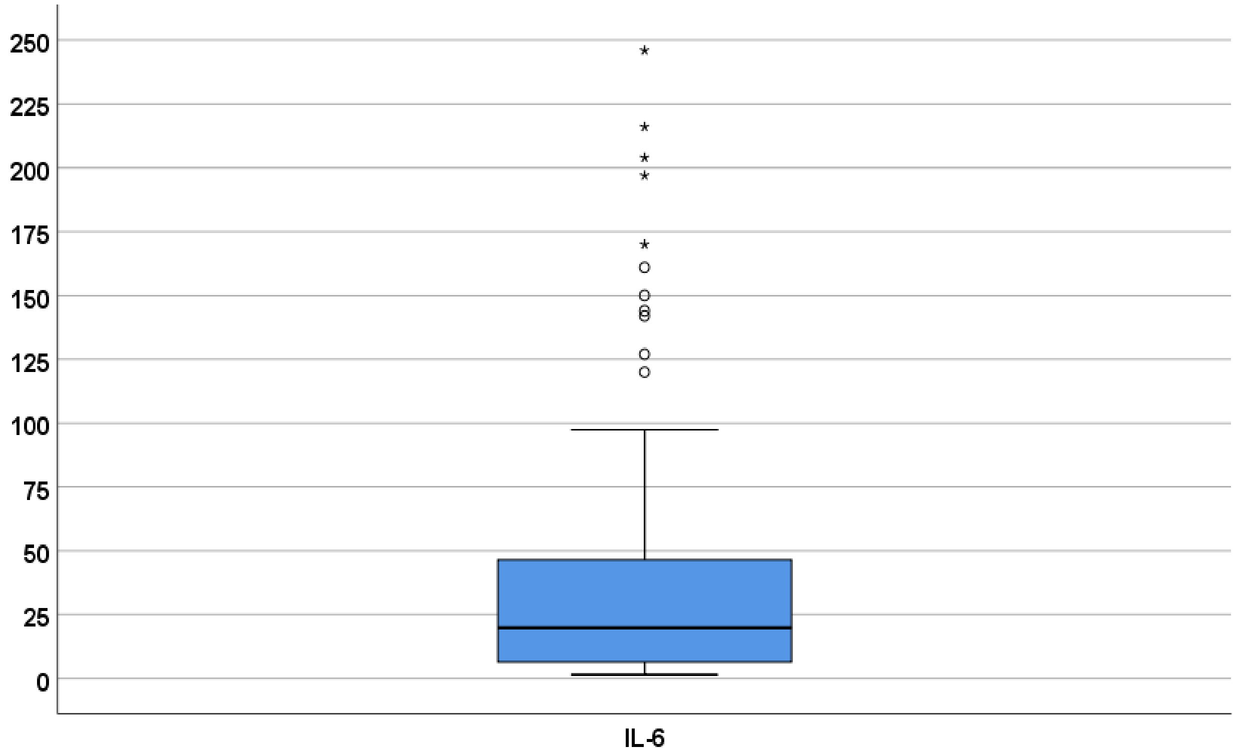

The circle is an indication of outlier; the asterisk is an indication that an extreme outlier.
